# Increasing IQ Test Scores and Decreasing *g*: The Flynn Effect and Decreasing Positive Manifold Strengths in Austria (2005–2018)

**DOI:** 10.3390/jintelligence12120130

**Published:** 2024-12-23

**Authors:** Denise Andrzejewski, Sandra Oberleiter, Marco Vetter, Jakob Pietschnig

**Affiliations:** 1 Department of Development and Educational Psychology, Faculty of Psychology, University of Vienna, 1010 Vienna, Austria; sandra.oberleiter@univie.ac.at (S.O.); marco.vetter@univie.ac.at (M.V.); jakob.pietschnig@univie.ac.at (J.P.); 2 Department of Psychology, School of Social Sciences, Heriot Watt University, Dubai P.O. Box 38103, United Arab Emirates; 3 Doctoral School of Cognition, Behavior, and Neuroscience (CoBeNe), University of Vienna, 1010 Vienna, Austria; 4 Department of Psychology, University of Salzburg, 5020 Salzburg, Austria; 5 Schuhfried GmbH, 2340 Mödling, Austria

**Keywords:** Flynn effect, positive manifold, psychometric *g*, ability differentiation, Austria

## Abstract

After almost a century of global generational IQ test score gains, the Flynn effect has, in the past decades, been observed to show stagnation and reversals in several countries. Tentative evidence from academic achievement data has suggested that these trajectory changes may be rooted in a decreasing strength of the positive manifold of intelligence due to increasing ability differentiation and specialization in the general population. Here, we provide direct evidence for generational IQ test score and positive manifold strength changes based on IQ test standardization data from 1392 Austrian residents between 2005 and 2018. Our analyses revealed positive Flynn effects across all domains of the IQ test (Cohen’s d from 0.21 to 0.91) but a trend toward decreasing strength in the positive manifold of intelligence (*R*^2^ from .908 to .892), though these changes were not statistically significant. Our results are consistent with the idea that increasingly inconsistent Flynn effect trajectories may be attributed to increasing ability differentiation and specialization in the general population over time.

## 1. Introduction

Generational IQ test score changes, known as the Flynn effect, yielded global increases of approximately two to four points per decade since the early twentieth century (Pietschnig and Voracek 2015). Gains have been observed to be about twice as strong in measures of fluid intelligence compared to crystallized intelligence and have generally been positive, irrespective of domain and country, throughout the 1900s (Flynn 1987; Pietschnig and Voracek 2015).

Flynn effect trajectories were observed to behave non-linearly across the twentieth century, with phases of rapid gains interspersed with periods of minimal increases and even virtual stagnation. However, by the late twentieth century, the previously observed seemingly continuous gains began to decrease in strength (Pietschnig and Voracek 2015), leading to speculations about a potential stagnation or reversal of this trend. Recent observations have indeed indicated less consistent Flynn effects, with several reports indicating deceleration (e.g., USA: Rindermann and Thompson 2013); stagnation (e.g., Australia: Cotton et al. 2005); or even reversals of test score gains (e.g., Norway: Bratsberg and Rogeberg 2018; USA: Dworak et al. 2023; Austria: Pietschnig and Gittler 2015).

Several candidate theories have been proposed to explain test score gains, with the most frequently cited ones including improved perinatal nutrition, access to medical services, and better schooling. While a stagnation of the Flynn effect may conceivably be attributed to diminishing returns and ceiling effects of IQ boosting factors, causes for a potential reversal of the Flynn effect are less clear. Some authors have suggested that the effects of selective reproduction patterns migration (Dutton and Lynn 2013), and mortality (Nyborg 2012) may be responsible for test score decreases, but direct empirical tests are inconsistent with these ideas (Pietschnig et al. 2018).

Therefore, it has been speculated that one possible reason for the seemingly erratic patterns in IQ change strengths and direction in recent accounts may be due to an examination of more fine-grained IQ domains (Oberleiter et al. 2024b). In other words, Flynn effect investigations of modern psychometric tests, which offer more in-depth evaluations of cognitive abilities, might be responsible for these inconsistent IQ trajectories to a certain extent. Previous Flynn effect studies were predominantly conceptually based on Cattell’s classic taxonomy, distinguishing between fluid, crystallized, and full-scale IQ. However, according to the currently most widely accepted intelligence theory, the Cattell–Horn–Carroll (CHC) model (Schneider and McGrew 2013), fluid and crystallized intelligence are merely two of several broad abilities. This means that a potential differentiation of IQ changes according to stratum II domains may have been masked by the comparatively crude assessment in past investigations.

In fact, recent Flynn effect findings based on stratum II domains from the CHC framework have yielded evidence for the differentiation of IQ trajectories across stratum II abilities in terms of both strength and direction. Specifically, some domains, such as comprehension knowledge, display positive changes, while others, like spatial orientation and working memory capacity, reveal negative trends. Moreover, some abilities, like processing speed, show no change (Lazaridis et al. 2022). Thus, to understand the underlying factors and causes driving these changes, it is necessary to examine trajectories in specific subdomains instead of higher-order scores.

Intriguingly, past observations have shown that IQ gains appear to be predominantly negatively associated with psychometric *g* (Must et al. 2003; Pietschnig and Voracek 2015; Woodley and Madison 2013; Woodley and Meisenberg 2013; but see Colom et al. 2001 for contrasting findings). Psychometric *g* can be understood as a consequence of the well-documented positive intercorrelations between virtually all intelligence (sub)tests. These positive intercorrelations are typically referred to as the positive manifold of intelligence. Conceivably, the observed negative association between the Flynn effect and psychometric *g* may indicate a weakening of the positive manifold due to increasing ability differentiation in the general population.

If this is indeed the case, we should be able to observe a cross-temporal decline of the positive manifold. Some initial support for this idea has been provided in observations of changes in average intercorrelations of WISC subtests in children (Lynn and Cooper 1993, 1994) and, more recently, in changes in the achievement *g* of Italian students (Pietschnig et al. 2023). However, direct evidence for positive manifold changes in intelligence is, so far, unavailable.

Importantly, test score changes and, consequently, changes in the positive manifold can only be meaningfully interpreted if the observed changes in test scores can be reliably attributed to population performance changes rather than item drift. Cross-temporal comparisons of test scores based on non-measurement invariant test instruments can yield misleading results. For instance, it was reported in 2015 that French IQ test performance decreased on the Wechsler Adult Intelligence Scale from 1999 to 2009 (Dutton and Lynn 2015). However, re-analyses of these data showed no evidence for a negative Flynn effect in France, with the observed decreases being due to differential item functioning between the examined cohorts (Gonthier and Grégoire 2022).

Here, we examined cross-temporal changes of the positive manifold between 2005 and 2018 in three standardization samples of a well-established intelligence test in Austria based on raw scores and measurement invariant latent means.

## 2. Materials and Methods

The present study was preregistered at the Open Science Framework (OSF) before accessing the data (https://osf.io/dsqth/).

### 2.1. Participants

This study accessed standardization samples of the Intelligence Base Functions (IBFs) from the database of a well-established Austrian test publisher. The sample comprised Austrian residents (*N* = 1392) aged 14 to 85 who participated in three test (re-)standardizations in 2005, 2011, and 2018. The sociodemographic sample characteristics are provided in Table 1.

### 2.2. Intelligence Base Functions (IBF; Blum et al. 2018)

The IBF is a 112-item cognitive assessment that measures intelligence based on an adapted version of the primary mental abilities (Thurstone and Thurstone 1941). It comprises four indices, namely numerical intelligence functions, verbal intelligence functions, spatial ability, and long-term memory. Numerical intelligence functions (NIF) are derived from two subscales, i.e., numerical reasoning and mathematical problem solving. Verbal intelligence functions consist of two subscales, i.e., verbal comprehension and verbal analogies. These indices assess the stratum II abilities of quantitative knowledge (Gq), comprehension knowledge (Gc), visual processing (Gv), and long-term storage and retrieval (Glr) according to CHC theory. In this study, we used raw score data of the six subscales from the standard version of this test. The IBF has shown satisfactory psychometric properties on the subscale level (α range [.85, .96]; (Schuhfried 2018)).

#### 2.2.1. Numerical Intelligence Functions

Numerical intelligence functions (NIFs) are assessed via two sections with 40 items and an average testing time of 25 min. The NIFs assess inductive reasoning using simple number sequence tasks (numerical reasoning) and calculation capacity with basic arithmetic text format tasks (mathematical problem solving) and can be understood as measures of the stratum II abilities fluid reasoning (Gf; numerical reasoning) and quantitative knowledge (Gq; mathematical problem solving).

##### Numerical Reasoning

In the first section (numerical reasoning, NR), test-takers must complete a number series task that involves identifying a logical pattern in a series of seven numbers and providing the following number in the sequence, for example, “384, 192, 96, 48, 24, 12, 6”.

##### Mathematical Problem Solving

In the second section (mathematical problem solving, MPS), test-takers are presented with arithmetic problems embedded in a text that involves basic computation and identifying relationships to solve the problem, for example, “4500 kg of brass contains 2700 kg of copper. How many kilograms of copper are in 900 kg of brass”?

#### 2.2.2. Spatial Ability

The Visualization (spatial ability, SA) subscale of the IBF assesses participants’ visualization and mental rotation abilities. It consists of 17 items and takes approximately 18 min to complete. Respondents are given a reference cube and six response alternatives and are asked which one of the responses represents a rotated version of the reference cube. The task requires the participants to form a mental representation of the reference cube, rotate it mentally, and compare it with the alternatives. It can be understood as a measure of the stratum II factor visual processing (Gv).

#### 2.2.3. Long-Term Memory

The long-term memory (LTM) subscale of the IBF includes two stages. In the first stage, 15 products must be memorized within seven minutes, including their brand names, price, and country of origin (e.g., nightgown, Levander, EUR 27.49, Denmark). The second stage takes place after a 20-min break, during which participants must answer questions about the memorized items in a restricted six-minute recall phase. For example, participants are given five choices to answer the following question: “One of the products priced at EUR 27.49 is made by …?”. The LTM domain of the IBF can be understood as a measure of learning efficiency (Gl) within the CHC framework.

#### 2.2.4. Verbal Intelligence Functions

The verbal intelligence functions evaluate verbal comprehension, which is understanding word meanings and applying them appropriately in a conversational context. This subtest contains 35 items, with a total working time of about 11 min, and it comprises two sections, namely verbal comprehension (VC) and verbal analogies (VAs).

##### Verbal Comprehension

In the first section, respondents are given a sentence completion task in which they must choose the correct word or phrase from a set of five alternatives. For example, for the sentence “A painting always includes a…”, participants are given the following response choices: (i) a frame, (ii) a canvas, (iii) a painter, (iv) a price, or (v) a museum. This task requires understanding word meanings and their contextual implications, which can be understood as a measure of the stratum II factor comprehensive knowledge (Gc).

##### Verbal Analogies

In the second section, respondents are shown three words, the first two of which have a certain relationship. The participants then must identify an analogous relationship between the third and a fourth word out of five response alternatives. For example, “Word: Sentence = Plate: ?” needs to be completed with either spoon, dish, crockery, hunger, or key. Verbal analogies is a of measure learning efficiency (Gl) within the CHC framework.

### 2.3. Procedure

The test and research center of a well-established Austrian test publisher (Schuhfried GmbH, Mödling, Austria), as well as partner universities and proctored online settings, were used between 2001 and 2018 to test all participants using a stratified sampling plan according to sex and age of the general population in Germanophone Central European countries. Specifically, age was stratified into 14 levels in five-year increments from 15 to 80+, using census data from Austria, Germany, and Switzerland (European Commission 2022) to ensure the distribution was representative of the population. Participants were all tested under the supervision of trained test administrators in standardized settings, participated in the study voluntarily, and received an hourly payment of about 9 USD for the time they spent participating. After completion, the participants were thanked and debriefed.

Data were collected in 2005, 2011, and 2018. For the 2005 cohort, data were collected between 2001 and 2007. Therefore, for our analyses, we assume a data collection year of 2005 for that cohort. Examinations after sampling have shown that the 2011 and 2018 IBF samples are population representative regarding age and sex but that the 2005 cohort is not (see Lazaridis et al. 2022).

### 2.4. Statistical Analyses

#### 2.4.1. Measurement Invariance

We assessed the measurement invariance of the IBF subtests across cohorts using multi-group confirmatory factor analyses (MGCFAs). This necessitates the availability of item-level data from all conditions. However, because no item-level data were available for the 2005 cohort, measurement invariance could only be examined between the 2011 and 2018 cohorts. We followed the procedure proposed by Wu and Estabrook (2016) by applying increasingly restrictive models to our data.

Typically, four levels of invariance are progressively established, namely configural, weak, strong, and strict invariance. However, for dichotomous data, a model with invariant thresholds and loadings is statistically equivalent to a baseline model (Wu and Estabrook 2016). Consequently, here, we first examined configural measurement invariance and subsequently compared the model fit with strict models. To establish strict invariance, group-equality constraints were applied to residual variances. Models were compared by inspecting Comparative Fit Indices (CFIs). The more restrictive model was adopted when the CFI changes between models did not exceed 0.01 (Cheung and Rensvold 2002). If the CFI changes between models were to exceed 0.01, we planned to establish partial invariance by iteratively freeing thresholds and variances (to establish partial configural invariance) or loadings (to establish partial strict invariance) of the items with the largest modification indices until the CFI differences fell below 0.01. Subsequently, we estimated latent means and variances.

#### 2.4.2. Positive Manifold Changes

To examine changes in the positive manifold, we applied a forced single-factor analysis (FA) using the subscales of the IBF. The explained variance (*R*-squared) of this model can be interpreted as a consequence of the positive manifold of intelligence, with larger *R*-squared values indicating a stronger positive manifold. Therefore, cross-cohort changes in *R*-squared scores can be interpreted as changes in the positive manifold of intelligence.

We further examined Cronbach’s α to assess cross-temporal changes in the g saturation of the IBF (see Revelle and Wilt 2013 for an overview). To this end, we treated all 112 items as belonging to a single scale. The idea of this approach rests on larger values as a consequence of a stronger cohesion between items. This is reasonable in the present analytical approach, because all items can be expected to contribute to the latent intelligence factor (psychometric *g*), with larger resulting α and ω_h_ values being indicative of a stronger positive manifold of intelligence. We analogously examined the changes in McDonald’s ω*_h_* (McDonald 1999), although the ω*_h_* were calculated on a subscale instead of on an item level. Changes in these indices can be attributed to changes in the intercorrelation of items and thus be interpreted as changes in the positive manifold of intelligence.

These analyses were performed for all three cohorts (i.e., 2005, 2011, and 2018). However, latent score-based analyses could only be conducted for the 2011 and 2018 cohorts due to the unavailability of item-level data for the 2005 cohort.

#### 2.4.3. Ability Tilt

We investigated changes in the distributions of ability tilt as a further analytical approach to examine changes in the positive manifold. Ability tilt describes asymmetries in the cognitive profiles of individuals, typically in terms of numerical and verbal test performance. In other words, ability tilt may be assumed to represent the relative strengths of individuals in verbal vs. numerical cognitive domains (Coyle 2014).

To calculate tilts, we followed the procedure outlined by Coyle (2014) by first standardizing the IBF’s numerical (NIF) and verbal (VIF) intelligence function subscale scores within each cohort. Then, within-subject differences between NIF and VIF scores were calculated by subtracting verbal scores from numerical scores. Positive tilt scores indicate a numerical tilt (larger numerical than verbal scores), whereas negative tilt scores represent a verbal tilt (larger verbal than numerical scores). Because tilts are calculated based on standardized values, within-cohort means are necessarily zero. However, in the presence of marked changes in the prevalence of asymmetries in the cognitive profiles of participants (e.g., because of a decreasing positive manifold strength due to increased ability differentiation), the shape of tilt distributions may be expected to change over time.

We calculated excess kurtosis values of these tilt scores for each cohort to examine potential changes in ability tilt distributions between cohorts. Positive excess kurtosis values describe a leptokurtic distribution with a sharper peak and fatter tails than a normal distribution. This indicates that ability profiles are more concentrated around the mean. In contrast, negative excess kurtosis values describe a platykurtic distribution with a flatter peak and thinner tails. Platykurtic distributions indicate that ability profiles are rather differentiated and asymmetric. Therefore, a weakening of the positive manifold should manifest in rather platykurtic distributions of ability profiles.

Moreover, we calculated the absolute mean ability tilt to further investigate asymmetries in cognitive profiles. The absolute mean ability tilt was determined by averaging the absolute value of the within-subject differences between NIF and VIF scores. This measure indicates the overall strength of the tilt, regardless of direction (numerical or verbal). By examining the absolute mean ability tilt, we aimed to quantify the extent of asymmetry in cognitive profiles (i.e., regardless of tilt direction). This approach allowed us to assess the general trend of ability differentiation within each cohort.

We used *R* (v4.3.2, R Core Team 2023) for all our analyses. Measurement invariance analyses were performed with the *lavaan* package (Rosseel 2012), and we used ggplot2 (Wickham 2016) to create figures. The *R*-syntax for our analyses is provided in the online Supplementary Materials.

## 3. Results

There was a positive Flynn effect in all IBF domains across the three cohorts (except a virtual stagnation for spatial ability from 2005 to 2011). Means and standard deviations for all IBF domains across all cohorts are provided in Table 2. However, performance change trajectories were differentiated in strength according to the cognitive domains of the IBF, with numerical reasoning showing the strongest gains from 2005 to 2018, followed by mathematical problem solving, verbal analogies, spatial abilities, long-term memory, and verbal comprehension. Pairwise comparisons of standardization samples revealed some variability in the standardized test score changes, with effect sizes ranging from trivial to large (Cohen’s *d* from −0.07 to 0.90). Both raw and latent mean scores showed consistent cross-temporal changes in terms of strength and direction. Table 3 presents the results from pairwise cohort comparisons for all subtests (results of the Flynn effect analyses are detailed elsewhere; see (Lazaridis et al. 2022)).

By means of two-way ANOVAs, we assessed the effects of data collection year (2007 vs. 2011 vs. 2018), sex, and their interaction on IBF performance across cognitive domains. The results indicate that data collection year had small to medium effects on all IBF subtests (*η_p_*^2^ range [.057; .108]). However, the sex and interaction effects were trivial in strength, with eta-squared values consistently below .01 (see Supplementary Materials Tables S1 and S2).

### 3.1. Measurement Invariance

Measurement invariance analyses showed that all of the six IBF subscales were invariant across all three cohorts (i.e., 2005, 2011, and 2018). The CFI changes did not exceed the 0.01 threshold between configural and strict invariance (CFI change range [<0.001, 0.005]). This indicates that performance changes are not attributable to item drift, and test score changes can be interpreted as meaningful population ability changes (measurement invariance fit statistics are detailed in Table 4). An example of the final measurement invariance model is provided in Supplementary Materials Figure S1.

### 3.2. Positive Manifold Changes

**Figure 1:**
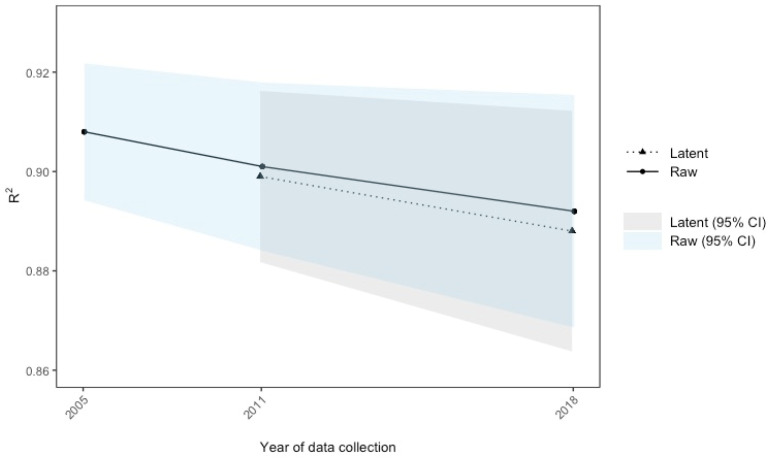
R^2^ values over time for the raw and latent scores.

Our analyses revealed that the *R*^2^ values consistently decreased over time from 2005 to 2018. In raw score-based analyses, *R*^2^ was .908 (95% CI [.894; .922]) in 2005, .901 (95% CI [.884; .918]) in 2011, and .892 (95% CI [.869; .915]) in 2018. Calculations based on the latent means showed decreasing *R*^2^ values from .899 (95% CI [.882; .916]) in 2011 to .888 95% CI [.864; .912]) in 2018. However, confidence intervals of *R*^2^ values overlapped, showing no nominally significant findings (see Figure 1 and Table 5).

Similarly, between-cohort comparisons of Cronbach’s α and McDonald’s ω_h_ showed consistently numerically smaller values in 2018 compared to the 2011 cohort (see Table 6), indicating a cross-temporally decreasing strength of the positive manifold in the IBF subscales. However, once again, confidence intervals overlapped, yielding no nominally significant differences with overlapping confidence intervals across both cohorts (Δα = .003 and Δωs = .003).

### 3.3. Ability Tilt

Skewness and kurtosis values were calculated for each cohort to describe the distributional characteristics of the data.

Examination of the changes in the excess kurtosis of ability tilts showed inconsistent patterns. The ability tilt values between 2005 (γ = 0.042) and 2011 (γ = −0.304) showed an increasingly platykurtic distribution (∆_γ_ = −0.346), indicating a higher prevalence of more asymmetric profiles in the latter cohort. However, from 2011 to 2018 (γ = 0.226), we observed an opposing trend towards a more leptokurtic distribution in both the raw score- (γ = −0.304 and 0.226, respectively; ∆_γ_ = 0.530), as well as latent mean-based analyses (γ = −0.428 and 0.045, respectively; ∆_γ_ = 0.473). The absolute means of the ability tilts showed a progressive increase across the three cohorts (absolute means = 0.598, 0.639, and 0.667 for 2005, 2011, and 2018, respectively), indicating, once again, evidence for a decreasing positive manifold strength (skewness, kurtosis, and absolute mean values for raw and latent ability tilts are provided in Supplementary Materials Table S3.

## 4. Discussion

Here, we examined (measurement invariant) changes in the positive manifold of intelligence in Austrian population representative standardization samples from 2005 to 2018. Our analyses provide tentative evidence for a decreasing positive manifold strength during a time of an ongoing positive Flynn effect in six subscales of a well-established intelligence test. These results present several points of interest, which we discuss below.

Our findings suggest an initial indication of a potential weakening in the intercorrelations among IQ subdomains from 2005 to 2018. Although the observed decrease in positive manifold strength is not statistically significant due to overlapping confidence intervals, the consistent downward trend across cohorts provides initial support for this pattern. This aligns with prior research indicating a negative relationship between the Flynn effect and psychometric *g* (Must et al. 2003; Pietschnig and Voracek 2015), pointing to a possible shift toward increasing cognitive differentiation and specialization. This interpretation is supported by evidence from recent comparisons of the original IBF standardization in 2007 with a purposefully recruited sample in 2024 that showed a significant decline in the positive manifold (Oberleiter et al. 2024a).

Because psychometric *g* results from the well-established positive manifold of intelligence, the negative correlation between *g* and test score gains may be seen as evidence that these associations are weakening. This may plausibly be a consequence of increased ability differentiation and specialization in the general population, which should manifest itself in more differentiation in intelligence test ability profiles. Our findings are in line with prior observations that have reported increasingly inconsistent Flynn effect trajectories, characterized by both positive and negative trends across various cognitive domains. For instance, studies have documented periods of deceleration, stagnation, or even reversals of IQ gains in several countries, such as Australia, Norway, and the USA, indicating variability in the trajectories of different cognitive abilities over time (e.g., Cotton et al. 2005; Bratsberg and Rogeberg 2018; Dworak et al. 2023).

Furthermore, emerging evidence suggests that the finer granularity of intelligence domains assessed under the CHC model may reveal differentiated patterns not observable in higher-level scores. For example, comprehension knowledge has shown positive changes, while abilities like spatial orientation and working memory have displayed negative trends (Lazaridis et al. 2022; Pietschnig and Gittler 2015). Conceivably, our findings may mean that ability differentiation is responsible for these inconsistent trajectories.

This idea can be thought of in terms of a decathlon. Performances in different decathlon disciplines (i.e., hurdling, shot putting, and high jumping) are typically positively intercorrelated, thus yielding a positive manifold of athletic performance and, consequently, an “athletics *g*”. In other words, individuals who excel in one discipline of the decathlon tend to perform well in others, indicating a general athleticism factor. The decathlon performance is the sum of the points awarded according to individual discipline’s performance. Intensive training in a specific discipline, for example, the high jump, consequently will not only lead to higher scores in this specific discipline but also in the overall decathlon performance. However, focusing intensely on one discipline may arguably affect scores of disciplines that are not focused on negatively. As long as the point increments that are gained in the focal disciplines are larger than the losses in the non-focal ones, the decathlon performance will increase. However, the same amount of training will eventually yield smaller performance gains in the focal discipline due to the law of diminishing returns (Spearman 1904). It can, therefore, be expected that increments that are lost will, at a certain point, match and eventually even outweigh the increments that are gained, thus leading to a decathlon performance decrease. In this case, the negative decathlon trajectory is not the result of a given decathlete having become a worse athlete but rather a consequence of increased specialization of this athlete (i.e., they have become a high jumper in our example; (Pietschnig et al. 2023)).

Intelligence test score changes can be thought about analogously, as long as we assume that specialization does not occur within a person but rather between generations. Our data are consistent with this idea by indicating a decline in the positive manifold while, simultaneously, test score increases are observed. These observations may be interpreted as evidence for increasing cognitive specialization of population members. In other words, the mean performance increase in all IBF subdomains appears to be driven by increasingly asymmetric individual cognitive profiles.

However, in light of the differing strengths of IQ gains between domains, it seems plausible that some specializations happen more often or substantially than others. For instance, we observed the strongest gains in numerical reasoning and mathematical problem solving. This is consistent with previous research showing larger gains in fluid IQ than crystallized IQ (Pietschnig and Voracek 2015). Recent studies, however, suggest differentiated patterns according to CHC model-based IQ subdomains, with some showing trivial gains or even reversals (Lazaridis et al. 2022; Colom et al. 2023; Oberleiter et al. 2024b). Arguably, modern educational practices may foster analytical and problem solving skills more substantially than other skills, as evidenced by the significant increase in participants with higher educational qualifications from 2005 to 2018.

Such a changing educational focus has been compared to “scientific spectacles” (Flynn 2010), which modern students are encouraged to do in modern educational environments. It has been argued that educational practices have led to a cognitive shift towards scientific and abstract thinking, where logical analysis, hypothesis testing, and systematic problem solving are focused on in teaching. Conceivably, the differential gains observed across various CHC model-based IQ subdomains may be related to educational and cultural emphases on certain cognitive skills (e.g., in STEM disciplines).

We also observed substantial gains in verbal analogies and moderate gains in verbal comprehension, echoing crystallized IQ trajectories observed in German-speaking countries (Pietschnig et al. 2010). Interestingly, we observed meaningful long-term memory gains, thus conforming to the findings of one previous study ((Rönnlund et al. 2013); but see (Gignac 2015) for stagnation and (Wongupparaj et al. 2017) for decreasing scores). Spatial ability trajectories were inconsistent across segments, yielding a virtual stagnation between 2005 and 2011 but moderate positive gains between 2011 and 2018. This mirrors similar prior observations for curvilinear trajectories for visual processing (Pietschnig and Gittler 2015; Lazaridis et al. 2022). Verbal comprehension yielded comparatively low gains, which conceivably could be a consequence of ceiling effects.

Our interpretation of a cross-temporally decreasing positive manifold strength is supported by our observation of decreasing variance explanations of single-factor analyses and reliability indices in our study. Decreasing *R*-squared values, as well as Cronbach’s alphas and hierarchical McDonald’s omegas, indicate decreasing intercorrelations of items and more heterogeneous response patterns, thus conforming to a weakening positive manifold.

Increasingly asymmetric tilt profiles, as evidenced by cross-temporally larger average tilt scores and more platykurtic tilt distributions (except for the 2011 to 2018 segment), are consistent with this interpretation. However, it needs to be acknowledged that findings from ability tilt calculations have been criticized for yielding potentially unreliable results in some instances (Sorjonen et al. 2023; but see Coyle 2023). While our findings reveal numerical trends that are consistent with increased ability differentiation, we acknowledge that they do not represent a direct test of ability differentiation as a cause for the Flynn effect. The observed patterns, such as the progressively increasing absolute mean of the ability tilts and the changes in kurtosis, align with the differentiation hypothesis, which suggests a possible shift toward more specialized cognitive profiles. However, given that the observed decreases in positive manifold strength are not statistically significant, we interpret these trends as merely tentative evidence for changes in *g* as a consequence of increasingly asymmetric ability profiles over time.

### 4.1. Potential Causes

The presently observed declining positive manifold strength suggests that cognitive profiles of general population members are becoming more differentiated due to increasing population ability differentiation. Conceivably, these results may be a consequence of changes in social priorities and demands that selectively reinforce the development of differentiated cognitive ability profiles in general and specific cognitive abilities in particular. For instance, it is possible that changes in education systems over time and modern technological developments have led to a shift in the types of cognitive abilities that are valued and reinforced. On the one hand, a stronger emphasis on standardized testing and math-centered curriculums in education and the extension of time people spend in educational systems may be expected to lead to increases, particularly in fluid ability domains. On the other hand, technological advances that have become an element of everyday life over the past century have been suggested to have altered our cognitive environment from one where problems were primarily dealt with on a concrete level to one where they are seen from an abstract or scientific perspective (Flynn 2010, 2019; Genovese 2002). These ideas may be assumed to be consistent with our observations of the strongest gains in our study having taken place in numerical reasoning and mathematical problem solving.

Specifically, over the twentieth century, educational curricula expanded considerably from the basic skills of reading, writing, and arithmetic to include a broad array of subjects, such as expanded science and math education (Flynn 2010). In this vein, educators have increasingly fostered abstract cognitive skills instead of concrete ones, favoring the abstract over the concrete. As educational curricula evolved, possibilities to specialize within comparatively early stages of educational training have become available (e.g., high school programs with a dedicated STEM focus). Increasing possibilities to specialize in certain training may incidentally lead to developing more specialized (i.e., differentiated) cognitive ability profiles and, thus, a decreasing positive manifold strength.

Technology has also been hypothesized to affect test score changes (e.g., Neisser 1997), even though the largest IQ gains were observed before 1980 when technology arguably did not permeate as many aspects of everyday life as it does today (Pietschnig 2016). However, it seems plausible that cultural shifts have led not only to new ways to work, play, communicate, and interact with each other but have also resulted in fundamental changes in cognitive skills needed for existence and participation in modern society (Clark et al. 2016). For instance, there has been an increased emphasis on technological literacy and digital skills in recent years.

The shift towards technological literacy and digital skills integration is illustrated by initiatives in many countries around the world and are often funded by governmental institutions. For instance, the Digital Skills Initiative of the European Union supports, amongst others, digital literacy education in schools across Europe (European Commission 2020). Similarly, the Digital Skills for Today’s Workforce Act seeks to bridge the digital divide in the U.S. by equipping workers with the skills needed to thrive in the modern job market (Coolberth 2024). Such programs are supported by significant investments in digital infrastructure on a governmental level and foster the development of domain-specific skills (e.g., coding and data analysis). It is likely that more insidious mechanisms besides such initiatives provide environmental reinforcement for the development of specific skill and ability profiles that continue to increase ability differentiation in the population. While technology might not necessarily account for substantial increments of the Flynn effect over the past century, it may have likely contributed to the emerging increasing ability differentiation in the general population.

### 4.2. Limitations and Future Directions

Some limitations need to be acknowledged when interpreting the present study. First, our results can, at the present point, not be generalized to countries other than Austria, because the Flynn effect is well known to have yielded country-specific trajectories, at least in terms of test score change strength in the past. Although a similar mechanism of ability differentiation is a root cause for the changing Flynn effect trajectories, it seems like a plausible candidate cause for observations in other countries, so direct evidence is needed. Additionally, our 2005 cohort data were collected over a seven-year period (2001–2007), which may introduce noise when studying generational trends. Because we relied on standardization and archival data, the collection timeline was outside our control and was determined by the original test publisher’s protocols. Although a shorter timeframe would be ideal for assessing trends like the Flynn effect, this limitation is inherent to the nature of archival data.

Second, some of the presently observed decadal IQ (DIQ) changes were, in some cases, implausibly large. Since DIQs depend on observed timespans, even small variations between samples will lead to relatively large DIQs, even with small effects. Therefore, the large DIQ values, especially between 2005 and 2011, should not be interpreted at face value.

Third, it is important to note that, although there is a numerical decrease in *R*-squared values across cohorts, overlapping confidence intervals indicate that these changes are not statistically significant. Therefore, although our findings appear to conform to the idea of increasing ability differentiation as a cause for the observed change patterns, the present results represent only tentative evidence for this idea.

Fourth, potential effects of ability differentiation on the Flynn effect could not be directly tested. Future research could address this limitation by employing longitudinal and cross-sectional studies designed to test causal links between environmental factors—such as educational and technological influences—and changes in intelligence conceptualizations.

Finally, other than the 2011 and 2018 cohorts, the 2005 cohort included in our analyses did not entirely represent the general population regarding sex and age. This may have affected the change estimations. However, even when limiting our interpretations to comparisons between sex- and age-representative cohort samples (i.e., 2011 and 2018), we observed almost virtually identical Flynn effects patterns regarding effect directions (but not strengths). It needs to be acknowledged that comparatively few participants with education levels 1, 2, and 5 were included in the present investigation. Targeted investigations of changes in specific educational strata would be useful in future research. In a different vein, future researchers may wish to examine influences of changes in the number of subtests that contribute to full-scale IQ calculations in intelligence test batteries such as the Wechsler tests on the Flynn effect strength between test revisions.

## 5. Conclusions

Here, we provide evidence for a positive Flynn effect but a decreasing strength of the positive manifold of intelligence in Austrian general population members from 2005 to 2018. These findings may likely be attributed to increasingly asymmetric individual cognitive profiles due to increasing ability differentiation and specialization. It can be speculated that the increasing ability differentiation may be due to environmental reinforcement (e.g., in terms of education or technology) of specialization.

## Figures and Tables

**Table 1:** Sample characteristics according to cohort.

	2005	2011	2018
*N*	630	476	286
Age			
Range	14–77	16–85	14–83
Mean	38.14	44.57	41.84
SD	13.45	17.86	16.30
Sex			
Men	313 (50%)	237 (50%)	129 (45%)
Women	317 (50%)	239 (50%)	157 (55%)
Education *			
Level 1	17 (2.7%)	8 (1.7%)	4 (1.4%)
Level 2	47 (7.5%)	87 (18.3%)	25 (8.7%)
Level 3	351 (55.7%)	228 (47.9%)	92 (32.2%)
Level 4	152 (24.1%)	106 (22.3%)	116 (40.6%)
Level 5	63 (10%)	47 (9.9%)	49 (17.1%)

Note. * Levels classified according to EU Standards: EU educational level 1 = no school-leaving qualification, EU educational level 2 = compulsory schooling or an intermediate secondary school but without completing vocational training, EU educational level 3 = completion of vocational training or a course at a technical college, EU educational level 4 = school-leaving qualification at university entrance level, and EU educational level 5 = university degree.

**Table 2:** Means and standard deviations across cohorts.

Domain	Cohorts					
	2005		2011		2018	
	*M*	*SD*	*M*	*SD*	*M*	*SD*
Numerical Reasoning (NR)	0.0 *	5.08	1.71	4.68	4.47	4.59
Mathematical Problem solving (MPS)	0.0 *	4.59	1.11	4.64	3.41	4.53
Spatial Ability (SA)	0.0 *	4.13	−0.29	4.13	2.89	4.61
Long-term Memory (LTM)	0.0 *	4.81	1.50	4.86	3.44	4.84
Verbal Comprehension (VC)	0.0 *	3.19	0.68	2.82	1.92	2.18
Verbal Fluency (VF)—VIB	0.0 *	5.12	1.31	4.94	4.04	4.26

* Reference mean is set to 0, hence all other means are relative to the year 2005; M denotes mean and SD standard deviation.

**Table 3:** Test score changes of pairwise cohort comparisons for raw and latent score changes reported in Cohen’s d and DIQ.

Domain	Stratum II Domain	Interval	Raw Scores		Latent Scores	
			*d*	*DIQ*	*d*	*DIQ*
Full-scale IQ		2005–2011	.26 ***	6.44	-	-
		2011–2018	.67 ***	14.42	.69 **	14.75
		2005–2018	.35 ***	8.70	-	-
Numerical Reasoning (NR)	Fluid reasoning (Gf)	2005–2011	.35 ***	8.69	-	-
		2011–2018	.59 ***	12.71	.59 ***	12.65
		2005–2018	.91 ***	10.45	-	-
Mathematical Problem solving (MPS)	Quantitative knowledge (Gq)	2005–2011	.26 ***	6.51	-	-
		2011–2018	.58 ***	12.42	.50 ***	10.64
		2005–2018	.83 ***	9.57	-	-
Spatial Ability (SA)—RV	Visual processing (Gv)	2005–2011	−.07	−1.78	-	-
		2011–2018	.74 ***	5.79	.73 ***	15.66
		2005–2018	.67 ***	7.77	-	-
Long-term Memory (LTM)	Learning efficiency (Gl)	2005–2011	.31 ***	7.77	-	-
		2011–2018	.40 ***	8.54	.40 ***	8.59
		2005–2018	.71 ***	8.23	-	-
Verbal Comprehension (VC)	Comprehension-knowledge (Gc)	2005–2011	.23 ***	5.63	-	-
		2011–2018	.47 ***	10.15	.49 ***	10.45
		2005–2018	.66 ***	7.60	-	-
Verbal Analogies (VA)	Learning efficiency (Gl)	2005–2011	.26 ***	6.51	-	-
		2011–2018	.58 ***	12.42	.59 ***	12.72
		2005–2018	.83 ***	9.57	-	-

Note: Annual changes in IQ per decade (DIQ) can be calculated with the following formula: DIQ (interval) = ((d × 15)/interval) × 10; ** *p* < .01, *** *p* < .001.

**Table 4:** Fit statistics for all subtests and measurement invariance levels for 2011 and 2018.

Model	*χ* ^2^	*df*	*p(χ* ^2^ *)*	*CFI*	Model Comp.	*∆χ^2^ (∆df)*	*∆CFI*	Decision
Numerical Reasoning (NR)								
M1: Overall	544.136	170	.001	.997	-			
M2: Configural	691.347	358	.157	.999	-			
M3: Strict	763.521	378	.002	.996	M2	72.174 (20)	.003	Accept
Mathematical Problem solving (MPS)								
M1: Overall	230.034	170	.001	.997	-			
M2: Configural	384.901	358	.157	.999	-			
M3: Strict	462.276	378	.002	.996	M2	77.375 (20)	.003	Accept
Spatial Ability (SA)								
M1: Overall	169.555	119	.002	.996	-			
M2: Configural	285.831	253	.076	.997	-			
M3: Strict	351.354	270	.001	.993	M2	65.523 (23)	.004	Accept
Long-term Memory (LTM)								
M1: Overall	549.884	170	<.001	.965	-			
M2: Configural	697.226	358	<.001	.968	-			
M3: Strict	759.602	378	<.001	.963	M2	62.376 (20)	.005	Accept
Verbal Comprehension (VC)								
M1: Overall	94.494	90	.352	.997	-			
M2: Configural	208.616	193	.210	.990	-			
M3: Strict	228.002	208	.163	.988	M2	19.386 (15)	.002	Accept
Verbal Fluency (VF)								
M1: Overall	144.092	152	.664	1.000	-			
M2: Configural	259.385	342	.995	1.000	-			
M3: Strict	297.461	340	.953	1.000	M2	38.076 (2)	<.001	Accept

Note: *N* = 762; group 1 *n* = 476; group 2 *n* = 286.

**Table 5:** Principal component analysis (PCA) for the raw and latent scores.

	Raw Scores		Latent Scores	
	*R* ^2^	95% CI	*R* ^2^	95% CI
2005	.908	[.894; .922]	-	-
2011	.901	[.884; .918]	.899	[.882; .916]
2018	.892	[.869; .915]	.888	[.864; .912]

Note: The shaded ribbons represent the 95% confidence intervals (CIs) for the R^2^ values of raw and latent scores over the respective years.

**Table 6:** Reliability indices for 2005, 2011, and 2018.

	Raw Scores			
	α	95% CI	ω	95% CI
2005	-	-	.81	[.77; .82]
2011	.961	[.956; .966]	.81	[.70; .85]
2018	.958	[.953; .963]	.79	[.74; .84]

## Data Availability

The data presented in this study are not publicly available due to privacy restrictions.

## Supplementary Materials

The following supporting information can be downloaded at https://www.mdpi.com/article/10.3390/jintelligence12120130/s1: Table S1: Descriptive statistics for IBF subdomains by cohort and sex; Table S2: Results of two-way ANOVA for intelligence subtests by year and sex; Table S3: Skewness, Kurtosis, and Absolute Means for raw and latent Ability Tilt; Figure S1: Exemplary representation of strict factorial invariance estimation between the 2011 (Panel A) and the 2018 samples (Panel B) using the IBF subscale verbal comprehension subscale (16 items).
