# Supplementary material for: Increasing IQ Test Scores and Decreasing g: The Flynn Effect and Decreasing Positive Manifold Strengths in Austria (2005–2018)
Source: J Intell. 2024 Dec 23;12(12):130. doi: 10.3390/jintelligence12120130 (PMC11676032; doi:10.3390/jintelligence12120130)
Supplement: Supplementary file 1 [file jintelligence-12-00130-s001.zip › jintelligence-3258130-supplementary.pdf]

**Table S1**
Descriptive statistics for IBF subdomains by cohort and sex.

| | 2007 | | | | | | 2011 | | | | | | 2018 | | | | | |
|---|---|---|---|---|---|---|---|---|---|---|---|---|---|---|---|---|---|---|
| | Women | | | Men | | | Women | | | Men | | | Women | | | Men | | |
| | *N* | *M* | *SD* | *N* | *M* | *SD* | *N* | *M* | *SD* | *N* | *M* | *SD* | *N* | *M* | *SD* | *N* | *M* | *SD* |
| | 317 | | | 313 | | | 229 | | | 237 | | | 157 | | | 129 | | |
| Numerical Reasoning (NR) | | 8.75 | 4.95 | | 7.23 | 5.10 | | 9.76 | 4.84 | | 9.65 | 4.53 | | 12.54 | 4.45 | | 12.38 | 4.78 |
| Mathematical Problem-solving (MPS) | | 9.53 | 4.64 | | 8.11 | 4.44 | | 9.81 | 4.79 | | 10.07 | 4.49 | | 12.38 | 4.64 | | 12.05 | 4.40 |
| Spatial Ability (SA) | | 5.55 | 4.22 | | 4.21 | 3.93 | | 4.48 | 4.18 | | 4.70 | 4.08 | | 7.80 | 4.47 | | 7.74 | 4.78 |
| Long-term Memory (LTM) | | 8.86 | 4.74 | | 8.03 | 4.86 | | 9.79 | 4.91 | | 10.11 | 4.81 | | 12.03 | 4.79 | | 11.71 | 4.91 |
| Verbal Comprehension (VC) | | 11.53 | 3.13 | | 11.03 | 3.23 | | 11.83 | 2.98 | | 12.10 | 2.65 | | 13.54 | 2.04 | | 12.79 | 2.29 |
| Verbal Analogies (VA) | | 9.76 | 5.08 | | 8.14 | 5.04 | | 10.08 | 5.01 | | 10.46 | 4.88 | | 13.44 | 3.94 | | 12.45 | 4.58 |

**Table S2**
Results of two-way ANOVA for intelligence subtests by year and sex.

| | | | *F* | *df* | *p* | *ηp2* |
|---|---|---|---|---|---|---|
| Numerical Reasoning (NR) | Fluid reasoning (Gf) | Year | 84.31 | 2 | <.001 | .108 |
| | | Sex | 4.72 | 1 | .030 | .003 |
| | | Year x sex | 3.63 | 2 | .027 | .005 |
| Mathematical Problem-solving (MPS) | Quantitative knowledge (Gq) | Year | 53.79 | 2 | <.001 | .072 |
| | | Sex | 3.66 | 1 | .056 | .003 |
| | | Year x sex | 4.74 | 2 | .009 | .007 |
| Spatial Ability (SA) - RV | Visual processing (Gv) | Year | 58.26 | 2 | <.001 | .078 |
| | | Sex | 2.64 | 1 | .104 | .002 |
| | | Year x sex | 5.22 | 2 | .006 | .007 |
| Long-term Memory (LTM) | Learning efficiency (Gl) | Year | 50.41 | 2 | <.001 | .068 |
| | | Sex | 1.05 | 1 | .306 | .001 |
| | | Year x sex | 1.93 | 2 | .146 | .003 |
| Verbal Comprehension (VC) | Comprehension-knowledge (Gc) | Year | 42.01 | 2 | <.001 | .057 |
| | | Sex | 3.98 | 1 | .046 | .003 |
| | | Year x sex | 3.55 | 2 | .029 | .005 |
| Verbal Analogies (VA) | Learning efficiency (Gl) | Year | 66.01 | 2 | <.001 | .087 |
| | | Sex | 7.24 | 1 | .007 | .005 |
| | | Year x sex | 5.77 | 2 | .003 | .008 |

**Table S3**

*Skewness, Kurtosis, and Absolute Means for raw and latent Ability Tilt*

| | Raw | | | Latent | | |
|---|---|---|---|---|---|---|
| | *Kurtosis* | *Skewness* | \|*M*\| | *Kurtosis* | *Skewness* | \|*M*\| |
| 2005 | 0.042 | 0.019 | 0.598 | - | - | - |
| 2011 | -0.346 | -0.108 | 0.639 | -0.427 | -0.089 | 0.655 |
| 2018 | 0.226 | 0.127 | 0.667 | 0.045 | 0.172 | 0.691 |

*Note.* |*M*| = absolute mean tilts

**Figure S1.** Exemplary representation of strict factorial invariance estimation between the 2011 (Panel A) and the 2018 samples (Panel B) using the IBF subscale verbal comprehension subscale (16 items).

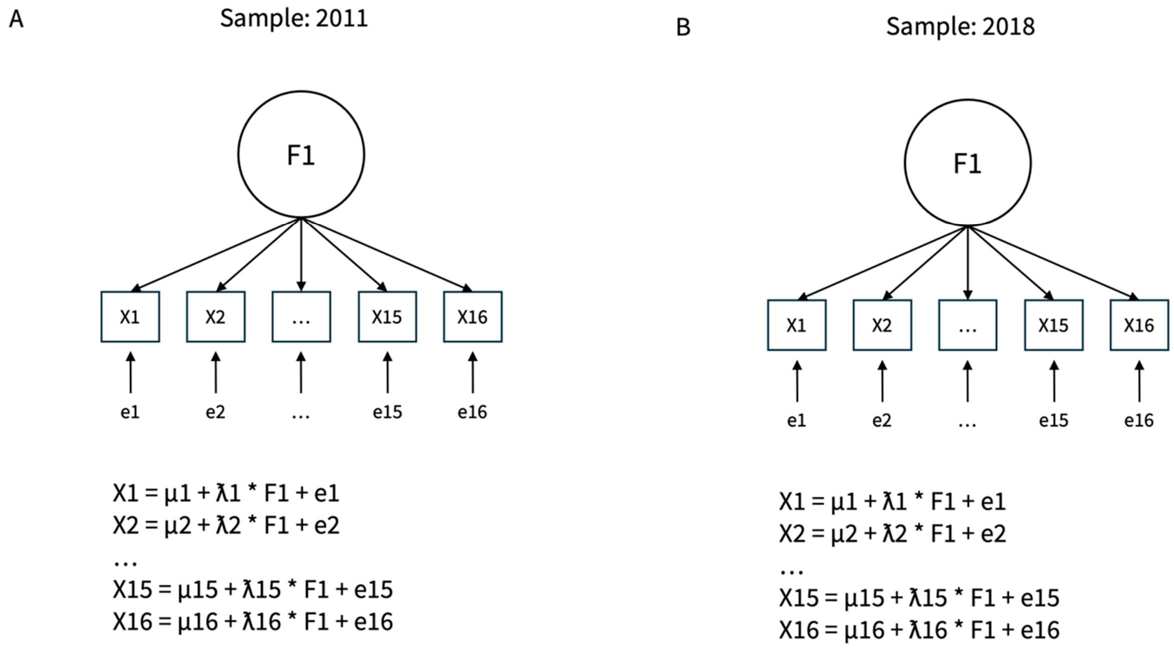


*Note.* 2011 sample: variance = fixed to 1, factor mean = fixed to 0; 2018 sample: freely estimated variance, free factor mean estimation.
